# HIV-exposed infants with EBV infection have a reduced persistence of the immune response to the HBV vaccine

**DOI:** 10.1186/s12981-021-00375-7

**Published:** 2021-08-04

**Authors:** Silvia Baroncelli, Clementina Maria Galluzzo, Giuseppe Liotta, Mauro Andreotti, Stefano Orlando, Fausto Ciccacci, Robert Mphwere, Richard Luhanga, Jean Baptiste Sagno, Roberta Amici, Maria Cristina Marazzi, Marina Giuliano

**Affiliations:** 1 National Center for Global Health, Istituto Superiore di Sanità, Viale Regina Elena, 299, 00161 Rome, Italy; 2 Department of Biomedicine and Prevention, University of Rome Tor Vergata, Via Montpellier, 1, 00133 Rome, Italy; 3 Saint Camillus International University of Health Sciences, Rome, Italy; 4 DREAM Program, Community of S. Egidio, P.O. Box 30355, Blantyre, Malawi; 5 Department of Human Sciences, LUMSA University, Via Traspontina 21, 00193 Rome, Italy

**Keywords:** HIV exposed uninfected infants, EBV acquisition, response to HBV vaccine, Malawi

## Abstract

**Background:**

In sub-Saharan African countries Epstein Barr virus (EBV) infection occurs in early childhood. We aim to investigate the factors associated with EBV acquisition and the impact of EBV infection on the humoral response to HBV vaccination in infants born from HIV-positive, antiretroviral-treated mothers in Malawi.

**Methods:**

A total of 149 HIV-exposed infants were included in this longitudinal study. EBV anti-VCA IgG were measured using an ELISA assay. The EBV seroconversion was correlated with the maternal viro-immunological conditions, with infant growth and immunological vulnerability, and with the humoral response to the HBV vaccine.

**Results:**

No infant was EBV-positive at 6 months (n. 52 tested). More than a third of infants (49/115 or 42.6 %) on study beyond 6 months seroconverted at 12 months. At 24 months, out of 66 tested infants, only 13 remained EBV-uninfected, while 53 (80.3 %) acquired EBV infection, rising the total proportion of EBV seroconversion to 88.7 % (102/115 infants). EBV seroconversion was significantly associated with a low maternal educational status but had no impact on infant growth or vulnerability to infections. Reduced HBsAb levels and accelerated waning of antibodies were associated with early EBV seroconversion.

**Conclusions:**

We found a heterogeneous timing of acquisition of EBV with the majority of infants born from HIV + mothers acquiring infection after 6 months. Anti-HBs levels were lower and appeared to wane faster in infants acquiring EBV infection.

## Background

The Epstein-Barr virus (EBV) is ubiquitous in human populations, it establishes a lifelong latency, with intermittent reactivation, and generally, limited clinical symptoms. In low-medium income settings, the peak of seroconversion occurs in the early years of life [1], whereas in developed countries the infection follows a bimodal distribution: infection acquisition can occur during the first year of life or can be delayed until adolescence [2]. EBV is a B-lymphotropic virus, associated with lymphoproliferative disorders including several important lymphomas [3, 4]. Soon after the infection, EBV acts reprogramming biological processes and pathways of B cells [5]. In children living in sub-Saharan regions, endemic to malaria and HIV (both linked to immune system dysfunctions), EBV infection can trigger substantial changes in the biology of the entire host B-cell compartment. The interaction between EBV infection and malaria is well recognized in the development of Burkitt lymphoma, one of the most prevalent childhood cancer in equatorial Africa [6]. On the other hand, early EBV infection in HIV-exposed children can concur in determining immune system dysfunctions; exploratory studies have indicated that early infection with EBV in infants is associated with reduced antibody responses to polysaccharides and measles vaccines [7], while others have shown that EBV latency can accelerate the decay of immune response to measles and rubella vaccines [8]. Limited is the information on the impact of early EBV infection on the magnitude and persistence of immune response to Hepatitis B (HBV) vaccination. Hepatitis B is highly prevalent in Malawi: in the general population HBsAg seroprevalence is estimated at about 7.9 % [9], and a similar rate has been observed in the HIV-positive population [10]. HBV vaccination is provided in Malawi as a component of a pentavalent vaccine preparation (scheduled at 6, 10, and 14 weeks of life), and the coverage of the 3-dose HBV vaccine is estimated to be around 95 % [11]. Nevertheless, the HBsAg prevalence in children (< 5 years) is estimated at around 1.5 % [9]. The vaccination schedule does not include the child birth dose.

In the last decade, the number of HIV-exposed but uninfected (HEU) infants has increased constantly; in Malawi, in 2019 their prevalence exceeded 7 % of the general child population, accounting for more than 600,000 children [12]. HEU infants show generalized immune system disorders leading to higher vulnerability to infections [13], impaired response to vaccinations (reviewed in 14), and a higher rate of mortality [15] compared to their unexposed counterparts. In this context, (of an immune vulnerable population) it is relevant to determine the rate and the timing of EBV infection, which could affect the maturation and the functions of the immune system over the life course.

In this study we evaluated EBV infection in a cohort of Malawian HEU infants with the following objectives: (a) to identify the potential factors associated with EBV infection acquisition in infants at 1 and 2 years of age, (b) to determine the possible impact of EBV infection on infants growth and vulnerability to infections and (c) to explore the impact of EBV infection on the magnitude of the humoral immune response following HBV vaccination and the persistence of protective titres over time.

## Materials and methods

### Study design

This sub-study included 149 infants, enrolled in a larger observational study that took place in Malawi between 2008 and 2011 to assess the safety and pharmacokinetics of maternal antiretroviral therapy administration during breastfeeding (Safe Milk for African Children[SMAC] study, [16]. In the study, treatment-naïve HIV-infected women started antiretroviral treatment (ART) during pregnancy and continued it until 6 months postpartum, or indefinitely if the baseline CD4 + count was less than 350/mm^3^. Women were instructed to exclusively breastfeed for up to 6 months. After delivery, the mothers and their infants were monthly visited for clinical and laboratory assessment for up to 24 months.

The longitudinal sub-study was conducted on a total of 115 infants with complete follow-up and available plasma/serum samples collected at 12 and 24 months.

To determine very early EBV acquisition, additional 52 infants were tested for EBV-IgG at 6 months of age. Eighteen of the 52 infants were also included in the longitudinal study (available samples at 12 and 24 months), while 34 had only the 6-month sample.

### Clinical assessments

Monthly clinic visits, scheduled as part of the cohort study, were performed during 24 months of follow-up. Physical examination was performed on mothers and infants and clinical data were recorded. Gastrointestinal and respiratory infections, and malaria, based on mothers’ reported symptoms, were diagnosed by clinical judgment following guidelines indication [17, 18].

### Laboratory assays

Cytomegalovirus (CMV) infection in infants was diagnosed by anti-CMV IgM antibody determination (at 1 month of age) or by real-time PCR for DNA (Enzygnost CMV IgM and kPCR PLX^®^ Cytomegalovirus (CMV) DNA Assay, Siemens Healthcare, Erlangen, Germany), at either 6 or 12 months of age, as already described [19].

Levels of antibodies elicited by HBV vaccine, scheduled at 6, 10, and 14 weeks of life, were determined by IgG anti-HBs assay (Enzygnost anti-HBs, Siemens Healthcare, Erlangen, Germany). According to previous studies, anti-HBs levels ≥ 10 mIU/ml were considered protective [20]. The maintenance of an adequate immune response to HBV was analyzed by measuring anti-HBs titres over time. The persistency of HBs titres was determined by their changes between 12 and 24 months; we arbitrarily categorized 4 types of dynamic responses to vaccine: (1) maintenance or increase of HBs IgG levels over 100 mIU/ml; (2) stable HBs IgG levels between > 10 < 100 mIU/ml; (3) clinically relevant decrease (> 50 %) of HBs IgG levels (4) poor response to HBV vaccination (HBs IgG < 10 mIU/ml at both time points).

Infant anthropometry variables were undertaken using methods already described [21]. Height-for-age (HAZ), weight-for-age (WAZ), weight-for-height (WHZ), and body-mass-index-for-age (BAZ) were calculated based on WHO references standard using WHO Anthro version 3.2.2 [22], using the internationally recognized z-scores and thresholds [23].

### EBV serology

Antibodies for viral capsid antigen VCA-immunoglobulin G (EBV VCA-IgG ELISA MyBioSource Inc, Southern California, San Diego USA) were measured using 10 µl of serum. The procedure was performed according to the manufacturer’s instructions. For each calibrator, negative and positive controls the mean of duplicate readings was used: only if the coefficient of variation for each duplicate was less than 15 % the test was considered valid. The OD was read at 450 nm within 15 min using an ELISA reader. The Ab Index of each determination was calculated by dividing the OD value of each sample by the cut-off (Calibrator OD × Calibrator Factor). The samples were considered EBV-positive when the Ab Index was greater than the cut-off value.

### Statistical analysis

The SPSS software, version 26 (IBM, Somers, NY, USA) was used for statistical analyses. Results are presented as medians with interquartile range (IQR) and percentages. Geometric mean titre was used for anti-HBs. Differences between groups were evaluated using the χ^2^ test or the Fisher’s exact test when appropriate for categorical variables, and by the Mann-Whitney *U* test for quantitative variables. The Wilcoxon test was used to detect longitudinal differences. Spearman’s correlation coefficient was used to evaluate correlations between quantitative variables. A linear regression analysis was performed to evaluate the determinants of EBV acquisition at Month 12, controlling for potential confounding factors (maternal age, viral load, CD4 + cell count, WHO stage, educational levels). Differences were considered statistically significant when P < 0.05.

## Results

### Patient characteristics

At enrollment, before antiretroviral treatment (ART), women had a median viral load of 4.1 HIV-RNA log Copies/ml (IQR: 3.3–4.6) and 338.5 CD4 + cell/µl (203.8–469.3). Based on their CD4 + cell count, women were introduced to antiretroviral therapy (a combination of stavudine (d4T), lamivudine (3TC), nevirapine (NVP), if baseline CD4 + count was < 350/mm^3^; or zidovudine (AZT), 3TC and NVP if baseline CD4 + count was > 350/mm^3^). During pregnancy, women received a median of 10 weeks (IQR: 7.0–13.0) of ART. At the time of delivery, most of the women had viremia levels below 1000 HIV-RNA copies/ml. Socioeconomic status was determined by educational status (64.3 %: no school or primary; 35.7 % secondary), occupation status (unemployed: 60.9 %), and presence or absence of electricity at home (no electricity: 78.3 %).

Most infants (97.4 %) were born by vaginal delivery. The median weight within 15 days from delivery was 3.2 Kg (IQR: 2.78–3.50). The male/female ratio was 54/61 (48/52 %).

### Anti-VCA IgG longitudinal study

A total of 52 infants were tested for anti-VCA IgG at 6 months. 45/52 (86.5 %) were negative to the IgG anti-VCA IgG test, and 7 infants were EBV positive. All 7 infants were subsequently re-tested and were negative for anti-VCA IgG, indicating maternal origin for these antibodies at 6 months.

At month 12, 49 out of 115 infants (42.6 %) were IgG anti-VCA positive. Fifty-three of the remaining 66 seronegative infants (80.3 %), developed an immune response against EBV in the following 12 months, as showed by the presence of IgG anti-VCA. Overall, at month 24 most infants (102/115, 88.7 %) had antibodies against EBV, and only 13 (11.3 %) were EBV-negative.

#### Maternal and infant factors influencing EBV infection acquisition

In the first 12 months, EBV infection acquisition in infants was not associated with maternal HIV parameters (WHO stage, p = 0.423, viral load, p = 0.779 CD4 + cell count, p = 0.655), nor with the duration (p = 1.000) or the type of regimen (p = 0.850) of antiretroviral treatment (Table 1), while it was significantly associated with lower socioeconomic conditions: 77.6 % of the mothers of infants who acquired EBV had poor educational level (vs. 54.5 % of the infants not EBV-infected at 12 months p = 0.018). In a linear regression analysis, adjusted for potentially confounding maternal variables (age, viro-immunological parameters, and ART duration), poor educational levels remained a significant determinant of EBV acquisition (p = 0.011).

**Table 1:** Analysis of potential maternal factors influencing EBV infection in infants during the first 12 months of life and infants characteristics

	EBV + infants (n = 49)	EBV− infants (n = 66)	P value
Maternal age (years)	28.0 (23.0–30.5)	26.5 (24.0–31.3)	0.847
Weight (kg)	59.5 (54.9–66.8)	55.5 (51.2–65.4)	0.049
Hemoglobin (g/l)	10.3 (9.4–11.3)	10.3 (9.1–11.4)	0.832
HBV infection (n,%)	3 (6.1 %)	4 (6.1 %)	1.000
CD4 + count (cells/µl)	390 (218–490)	336 (204–468)	0.655
HIV-RNA (logcopies/ml)	4.08 (3.26–4.68)	4.10 (3.28–4.60)	0.779
WHO stage (I/II/III–IV) (%)	77.6/10.2/12.2	77.3/19.7/3.0	0.423
ART duration in pregnancy (weeks)	10 (7.0–13.0)	10 (6.0–13.3)	1.000
ART regimen: (n, %)			
d4T- 3TC-NVP AZT-3TC-NVP	26 (53.1 %) 23 (46.9 %)	37 (56.1 %) 29 (43.9 %)	0.850
Unemployment (n, %)	32 (65.3 %)	38 (57.6 %)	0.444
Low educational level(no school or only primary) (n,%)	38 (77.6 %	36 (54.5 %)	**0.018**
No electricity at home (n, %)	41 (83.7 %)	49 (74.2 %)	0.260
Vaginal delivery (n, %)	47 (97.9)	64 (97)	1.000
Infants weight at birth (Kg)^a^	3.2 (2.8–3.5)	3.2 (2.6–3.5)	0.562
Male/female ratio (%)	49/51	45.5/54.5	0.850
HIV positive infants (n, %)^b^	1 (2.0)	1 (1.5)	1.000
CMV + infants at 12 months^c^	12/20 (60 %)	25/35 (71.4 %)	0.551

Values are expressed as median (IQR) or percentage

*ART* antiretroviral therapy, *d4T* stavudine, *3TC* lamivudine, *NVP* nevirapine, *AZT* zidovudine

^a^Weight measured within the first 15 days of birth

^b^Infants acquiring HIV infection during the first year of life

^c^Number of infants = 55

A high percentage of infants (37 of the 55 infants tested) acquired CMV infection during the first 12 months of life. The CMV infection occurred in 12/20 (60.0 %) infants EBV + and in 25/35 (71.4 %) EBV− infants (p = 0.551, Table 1).

Infants’ birth weight, gender, and body mass index at 1, 6, and 12 months were not associated with an increased infants’ vulnerability to EBV infection at 12 months. EBV infection was observed in 1 of the two cases of pediatric HIV (HIV infection at 3 months), while the other HIV + infant (HIV infection at 12 months) remained EBV-negative during the study.

#### Impact of EBV infection

At 24 months the 13 infants remaining EBV-seronegative did not differ from those who acquired the infection; no differences were found in male/female ratio, growing rate (BMI and malnutrition indices at different time points, data not shown), the occurrence of other infections. During the 24 months of follow-up, 67.8 % of the infants experienced at least one of the most common infections reported in Malawi: gastroenteritis, respiratory infections, and malaria. Based on the timing of EBV infection acquisition, the prevalence of cumulative low respiratory tract infections (18.4.0 %, 28.3 and 15.4 %, p = 0.392), malaria (40.8 %, 43.4 and 69.2 %, p = 0.175) and gastroenterological events (34.7 %, 34.0 and 53.8 %, p = 0.925) did not differ between the infants that EBV-seroconverted at 12 months (n = 49), within 24 months (n = 53) and those remaining seronegative (n = 13) during the study.

The magnitude and the persistence of the immune response to HBV vaccination were measured in all infants by determining anti-HBs IgG titres. At 12 months anti-HBs concentrations did not differ in infants according to the presence of EBV infection (geometric mean EBV+: 101.7 ± 51.6 vs. EBV−:138.4 ± 42.9 mIU/ml, p = 0.777). Anti-HBs IgG titres over 10 mIU/ml (considered protective) were observed in 87.2 % of EBV + and 92.2 % of EBV− infants, p = 0.523.

**Fig. 1:**
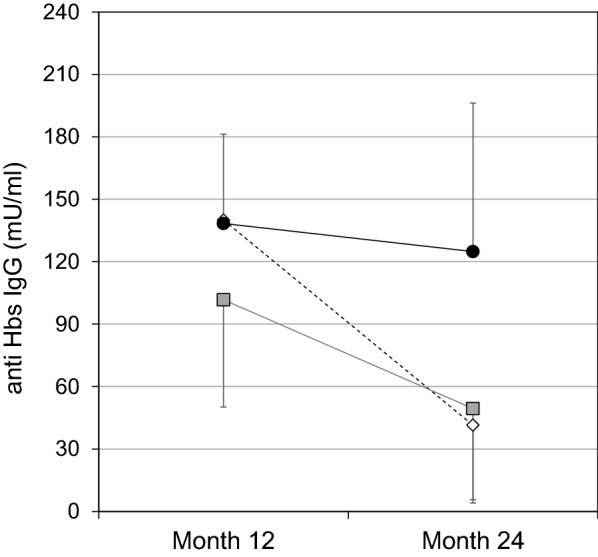
Longitudinal changes of anti-HBs IgG serum levels (geometric mean + SE ) in the 12- and 24-months old infants: EBV seropositive infants to anti-VCA IgG from month 12 (n = 47, grey square); EBV seronegative at month 12 (n = 64) that seroconverted within month 24 (n = 51, white diamond) and those that remained anti-VCA IgG negative (n = 13, black circle)

At month 24 the anti-HBs titres were 124.8 ± 71.4 mIU/ml in EBV-seronegative infants, and 41.4 ± 45.4 mIU/ml and 49.4 ± 35.8 mIU/ml in infants who seroconverted at 12 months or at 24 months, respectively (intergroup difference p = 0.008, Fig. 1).

Generally, the longitudinal trend in anti-HBs IgG titres showed a decline in all infants which became statistically significant only in the EBV seropositive infants, independently by the time of seroconversion (EBV-free: p = 0.070; EBV + month 12: p = 0.004, EBV + month 24: p < 0.0001, Fig. 1). A more detailed description of the evolution of anti-HBs levels during 12–24 months is reported in Table 2. The proportion of infants with high (> 100 IU/ml) antibody levels is underrepresented (15/52, 28.8 %) in infants that became infected during the second year of life, compared to infants with early infection (21/45, 46.7 %) and EBV-negative infants (7/11, 63.6 %). Importantly, more than 50 % (27/52) of infants with EBV acquisition during the second year of life experienced a relevant decay of vaccine-specific anti-HBs Ab. At month 24, 82.7 % of EBV-seropositive and 100 % of EBV-seronegative infants had anti-HBs titres > 10 mIU/ml.

**Table 2:** Longitudinal evolution of anti-HBs levels after HBV vaccination in infants that acquired EBV infection within 12 or 24 months or remained EBV negative

Month 12		EBV +		EBV−	P values
Number pz	n	47		64	
Anti-HBs IgG	(mlU/ml)	101.7 ± 51.8		138.4 + 42.9	0.777
Infants with anti-HBs > 10 mIU/ml	(n, %)	41 (87.2 %)		60 (92.2 %)	0.523

Month 24		EBV + at month 12	EBV + at month 24	EBV−	
Number pz	n	45	52	11	
Anti-HBs IgG	(mlU/ml)	41.4 ± 45.4	49.4 ± 35.8	128.4 ± 71.4	0.139
Infants with anti-HBs > 10 mIU/ml	(n, %)	36 (78.3 %)	45 (86.5 %)	11 (100 %)	0.154
Anti-HBs evolution over time					
Maintainance or increase > 100mlU/ml	n, (%)	21 (46.7 %)	15 (28.8 %)	7 (63.6.0 %)	0.039
Stable levels > 10 < 100 mlU/ml	n, (%)	3 (6.7 %)	4 (7.7 %)	1 (9.1 %)	
Relevant decrease > 50 %	n, (%)	14 (31.0 %)	27 (51.9 %)	3 (27.3 %)	
Poor response < 10 mlU/ml	n, (%)	7 (15.6 %)	6 (11.5 %)	0 (0.0 %)	

The persistency of anti-HBs titres was arbitrarily categorized into 4 types of dynamic responses to the vaccine

## Discussion

In this study, we confirm that Epstein Barr Virus infection is common in early childhood in Malawi. In our cohort, EBV acquisition was not associated with the viro-immunological maternal conditions, while disadvantaged social conditions seemed to favor earlier EBV infection. EBV seroconversion did not seem to influence the infant’s growth and the vulnerability to other infections, but our data suggest that EBV infection could have an impact on the persistence of the immune response to HBV vaccination.

More than 90 % of the global population in adult age is EBV-infected [24]. Here we report a 42.6 % rate of EBV seroconversion in the first 12 months, rising to 88.7 % of the total 115 HEU infants at 24 months. The rates and dynamics of EBV acquisition we found were comparable to data from previous studies (which found seroprevalences between 40 and 47 % at 12 months), conducted in geographical regions (Kenia, Uganda, and Ghana) [25–27] that share analogous socioeconomic and environmental features of Malawi; similar dynamics of EBV infection has been observed even in the pre-HIV epidemic era [28], suggesting that HIV infection could have a marginal role in the early acquisition of EBV infection. In this view, we did not found any association between EBV seroconversion and maternal viro-immunological conditions.

EBV acquisition can occur in the first few months of life (1–6 months of age) when the maternal immunological conditions are severely compromised by infections such as malaria and HIV which can affect the placental transfer of adequate levels of protective antibodies [1, 25, 29–31]. In our study EBV seroconversion was not observed before 6 months; although only 52 infants were tested at 6 months of age, and no one had their own anti-VCA antibodies (in few cases they were of maternal origins, as confirmed by seronegative tests afterward), and including the other infants still seronegative at 12 months (66 out of 115), we are confident in saying that EBV infection was not acquired during the breastfeeding period. These findings are in agreement with previous studies [26, 33] reporting that the improved health maternal conditions (antenatal ART coverage and ART continuation in the breastfeeding period) may have an impact on the delayed onset of EBV infection.

A significant risk factor for EBV seroconversion in the first year of age was related to social disadvantages; although our study was not designed to examine direct markers of socioeconomic status, the mother’s educational level was a contributing factor for EBV seroconversion during the first years of life. Similar results have been observed by others [33, 34], highlighting that early EBV infection can vary globally from 20 to 80 % depending on ethnic, geographical, and socioeconomic determinants [35].

Such as EBV infection, CMV infection typically occurs earlier in sub-Saharan Africa than in western countries [36]; in our study, 67.3 % of infants acquired CMV infection during the first years of life. Many studies reported that the interactions between CMV and EBV co-infection can affect immune function and response to vaccination, both in children and adults [37–39]. In this study, we were not able to assess the possible synergic impact of CMV coinfection on humoral response, because of the limited number of infants with available CMV tests (n = 55).

In contrast with a recent study [27], we did not found a prevalence of abnormal growth in EBV-infected infants, and the EBV seroconversion was not associated with increased vulnerability to infections. However, we found interesting data on the immune response to HBV vaccination. As already observed, HEU infants are capable of mounting a sufficient immune response for HBV vaccination to confer adequate protection [14, 40]. In the present study, at month 12, a cumulative percentage of 90.1 % of HEU infants had adequate anti-HBs IgG levels, which declined significantly at month 24, as already observed in a previous study on the same cohort [40]. Here we found that the EBV infection seems to influence the persistence of anti-HBs titres over time. At 12 months EBV-seronegative infants presented a 25 % higher level of anti-HBs than their EBV-seropositive counterparts. Then, anti-HBs levels drastically declined in infants who EBV seroconverted in the following 12 months, while remained quite stable in the few infants that remained EBV-seronegative. The very limited number of infants belonging to the former group allows us only to speculate on the potential impact of EBV on functional responses of B-cells [41]. Pediatric studies reported reduced serological responses to vaccination to measles, rubella, and meningococcal polysaccharides (MPV) in EBV-positive children from different cohorts [7, 8] suggesting that the timing of EBV infection could affect the immunological response to vaccines by the rapid decay of adequate serological levels of protection. These data are consistent with EBV biology; the infection triggers important changes in the B cells by reprogramming biological processes, inducing mutations that may interfere with antibody production [5, 42].

This study has some important limitations. The limited number of infants and the high rate of EBV seroconversion in the first 2 years of life did not allow us to have sufficient statistical power to perform a more sophisticated analysis between EBV positive and negative children. Moreover, we have assumed positive anti-VCA IgG as the result of EBV infection and did not perform other serological tests nor confirmatory EBV-DNA determination, which could have allowed us to monitor the host control of EBV infection. However, anti-VCA antibodies levels remain relatively stable throughout the life of the host [30] and can be considered a good surrogate to detect EBV acquisition. Another limit of our study is the lack of information regarding laboratory-confirmed malaria (although our study took place in an endemic area for *Plasmodium falciparum)* since malaria is considered a strong predictor of early EBV primary infection [6, 31].

## Conclusions

In conclusion, here we confirm that EBV acquisition in Malawian HEU infants occurs mostly during the first two years of life, and we suggest that the onset of infection can be delayed after 6 months of age, in the presence of improved immunological conditions of their HIV + ART-treated mothers. EBV acquisition does not seem to have an impact on children’s growth nor to increase their vulnerability to infections but infants with EBV acquisition have a reduced persistence of the immunological response to HBV vaccination, possibly compromising the long-term protection against hepatitis B infection. In this view, surveillance programs should be activated to monitor the serological persistency after vaccinations in areas endemic for early EBV infection, and possibly, re-schedule vaccination protocols, to confer lifelong adequate protection.

## Data Availability

The data that support the findings of this study are available from the corresponding author, (SB), upon reasonable request.

## Abbreviations

HEU: HIV exposed uninfected
EBV: Epstein Barr Virus
ART: Antiretroviral treatment
